# Response to the Letter to the Editor: Recalibrating evidence: a cautionary note on the use of post hoc thresholds in IPD meta-analyses

**DOI:** 10.1530/ETJ-25-0323

**Published:** 2025-11-26

**Authors:** Franz Sesti, Tiziana Feola, Pasquale Dolce, Valentina Guarnotta, Alessandro Veresani, Elia Guadagno, Filomena Bottiglieri, Maria Grazia Tarsitano, Mauro Salducci, Andrea M Isidori, Annamaria Colao, Antongiulio Faggiano, Elisa Giannetta, on behalf of NIKE group

**Affiliations:** ^1^ Department of Experimental Medicine, Sapienza University of Rome, Rome, Italy; ^2^ Neuroendocrinology, Neuromed Institute, IRCCS, Pozzilli, Italy; ^3^ Department of Translational Medical Science, Federico II University of Naples, Naples, Italy; ^4^ Department of Health Promotion, Mother and Child Care, Internal Medicine and Medical Specialties, Section of Endocrinology, University of Palermo, Palermo, Italy; ^5^ Endocrinology Unit, Department of Internal Medicine and Medical Specialties (DiMI), University of Genoa, Genoa, Italy; ^6^ Department of Neurosciences, Reproductive Sciences and Dentistry, Gynecology Unit, Federico II University Hospital of Naples, Naples, Italy; ^7^ Endocrinology, Diabetology and Andrology Unit, Department of Clinical Medicine and Surgery, Federico II University of Naples, Naples, Italy; ^8^ Department of Medical and Surgical Science, University Magna Grecia, Catanzaro, Italy; ^9^ Departiment of Organ Senses, Sapienza University of Rome, Italy; ^10^ Centre for Rare Diseases (ENDO-ERN accredited), Policlinico Umberto I, Rome, Italy; ^11^ Endocrinology Unit, Department of Clinical and Molecular Medicine, Sant’Andrea Hospital, ENETS Center of Excellence, Sapienza University of Rome, Rome, Italy

Dear Editor,

We are grateful to the author of the letter (1) for his interest in our recent paper, ‘Role of the calcium stimulation test in diagnosing medullary thyroid cancer: is it adequate to achieve a diagnosis in both sexes? An individual patient data meta-analysis’ (2) in the European Thyroid Journal (2025), and for his comments on the methodological aspects of our IPD meta-analysis. We would like to clarify our methodological choices and address the concerns raised.

Regarding the absence of PROSPERO registration, we recognize that registration would have improved transparency. We defined the analytic framework and objectives before conducting the analyses at the ‘NIKE’ (Neuroendocrine tumors Innovation Knowledge and Education) workshop held by Prof. Annamaria Colao, Prof. Antongiulio Faggiano, and Prof. Andrea M Isidori. The aim of the workshop was to boost the advancement of knowledge in various aspects of neuroendocrinology. However, we recognize the value of protocol registration to limit potential bias. This limitation was acknowledged in our paper, and we agree that future research should adopt protocol registration as a standard practice.

We agree that threshold selection is an important issue in diagnostic accuracy studies and that imposing a single data-derived threshold may eliminate natural threshold heterogeneity. However, the use of different cutoffs across primary studies may not be clinically meaningful. Our analyses are exploratory in nature, and the same threshold was applied across all studies to obtain consistent performance estimates and a global optimal cutoff value that could be uniformly applied. Using a unique threshold ensures clinical interpretability, whereas allowing study-specific thresholds could lead to loss of comparability and clinical relevance. In this context, we would like to clarify the rationale and context behind our approach.

Considering the considerable variability in thresholds across the studies included in our analysis, from as low as 79 pg/mL (3) to as high as 1,620 pg/mL (4), we decided that a harmonized thresholding strategy was required to facilitate meaningful pooling and comparison of diagnostic accuracy metrics. Sex-specific cutoffs were derived based on biological rationale (5) and supported by exploratory analyses (3, 4, 6, 7, 8, 9, 10). We do acknowledge that, while our sample size was adequate for exploratory modeling, it limits the robustness and generalizability of the thresholds derived. However, consensus does not currently exist for definitive sex-specific basal or stimulated threshold values that reliably and consistently distinguish medullary thyroid carcinoma from other clinical conditions. Thus, our meta-analysis contributes to that practical, unresolved clinical question.

We acknowledge that implementing uniform thresholds in heterogeneous studies may diminish the observed between-study variance. However, we aimed at isolating the test’s performance under a fixed decision rule. Our goal was to attempt to strike the proper balance between methodological rigor and clinical significance within a gray area of oncological endocrinology. We acknowledge that diagnostic thresholds often differ due to local practices, institutional preferences, or assay variability, rather than underlying biological necessity.

The absence of internal or external validation techniques is a valid point. We do agree that such validation methods would have added robustness to our estimated threshold, but we acknowledge this as a limitation. We will therefore include a formal internal validation procedure in our future work.

Ultimately, our intention was not to replace existing thresholds used in individual studies but to explore the potential utility of standardized sex-specific cutoffs within the framework of IPD. We appreciate the constructive nature of the critique and agree that further work in larger prospectively harmonized datasets is needed to validate these thresholds.

We acknowledge that, according to QUADAS-2 guidance, the absence of pre-specified diagnostic thresholds may indicate a high risk of bias in this domain. However, we respectfully note that many of the studies included were explicitly designed to discover optimal thresholds. Their intent was exploratory, not confirmatory, and in these scenarios, thresholds are not pre-specified but data-driven. We fully acknowledge that this study design choice can limit the generalizability of the findings; nevertheless, we argue that it should not imply a high risk of bias, as the exploratory nature of the threshold derivation is clearly stated and justified in the methods section of the original study.

We fully acknowledge the presence of clinical and methodological heterogeneity within included studies, particularly among study populations and diagnostic thresholds. Although both the fixed-effects and random-effects models were initially employed as a way of testing the robustness of our pooled estimates, we agree that, given the heterogeneity across studies, the use of random-effects models would have been more appropriate as the default approach. Our intent was not to use these models interchangeably but rather to assess the consistency of findings across different assumptions regarding between-study variability. For this reason, the primary summary estimates, including the pooled AUCs and diagnostic accuracy metrics, were derived using random-effects models, which we agree are more appropriate under such conditions. Although we applied fixed-effects models in an exploratory capacity to examine the sensitivity of our results given different assumptions, it is important to reiterate that our final and default approach for our final analysis was random-effects models, which we agree are the default approach for heterogeneity of this nature. Notably, using fixed-effects and random-effects models gave identical results when checking diagnostic accuracy in males, suggesting minimal between-study heterogeneity for that specific analysis and supporting the robustness of the findings.

We agree that heterogeneity in diagnostic test accuracy studies is multifactorial and frequently related to threshold effects, spectrum variation, and correlated sensitivity/specificity patterns. We acknowledge that *I*^2^ statistic for sensitivity and specificity may not account for threshold-related variation or the correlation between these parameters, thus potentially leading to an overestimation of heterogeneity. For this reason, we reported *I*^2^ for the AUC and not for sensitivity and specificity. However, we are also aware that computing *I*^2^ for the AUC has methodological limitations, as it is derived from the automated analyses done by the R Shiny application IPDmada software (11), which computes and reports *I*^2^ values for AUCs by default. Thus, it is important to note that the *I*^2^ results presented here should be considered a secondary exploratory analysis and should be interpreted with caution, given the limitations of the default implementation of the software. Regarding the interpretive cutoffs of *I*^2^, even though our thresholds differ from those suggested in the Cochrane Handbook, the overall interpretation of our findings remains unchanged. In our analysis, the *I*^2^ statistic was used descriptively, to give readers a general indication of the variability across studies. The thresholds we used – low (<25%), moderate (25–50%), and high (>50%) – were based on the widely cited work by Higgins *et al.* (12), where these ranges were originally proposed as a general interpretive guide. We acknowledge, however, that the Cochrane DTA guidance recommends a more cautious and context-specific interpretation of *I*^2^ in diagnostic accuracy reviews and that heterogeneity in this setting is often structural as opposed to simply statistical. We also acknowledge the point that *I*^2^ is not ideal for composite metrics, such as the AUC, which do not account for the trade-off between sensitivity and specificity across thresholds. We used random-effects models, as we expected heterogeneity, and conducted sensitivity analyses to explore the robustness of our findings. We reported AUCs, as we were trying to provide an interpretable summary of diagnostic performance by sex, given the clinical relevance of this distinction. However, we agree that pooled IPD-derived receiver operating characteristic (ROC) curves (Figs 3 and 5 in (2)) provided a better overall representation, as they preserve the full distribution of test characteristics.

Although bivariate or HSROC models are the preferred option for evaluating test accuracy in a meta-analysis, we were unable to use these models because some of the included studies did not report paired sensitivity and specificity data for participants enrolled in the same study. Therefore, we constructed pooled 2 × 2 tables from IPD using the derived thresholds to provide intuitive summaries of test performance by sex, using the same thresholds applied across the pooled IPD.

Regarding the data and code availability, we recognize the importance of enabling reproducibility; however, the raw IPD cannot be shared publicly due to institutional and ethical restrictions.

Regarding the observation of the meta-analysis of AUCs, we agree with the author that this type of meta-analysis is not a methodologically appropriate meta-analysis design for diagnostic accuracy studies. We recognize that aggregating AUC values derived from individual studies is not the most appropriate approach and that the resulting heterogeneity metrics should therefore be interpreted with caution. We also concur with the concern raised about confidence intervals exceeding the theoretical maximum value of 1.00. This issue likely stems from the lack of a logit transformation before meta-analytical modeling, as correctly noted by the author. We apologize for this oversight.

As previously indicated, the analysis was carried out using IPDmada. Unfortunately, this software performs AUC pooling automatically and does not allow users to view or modify the underlying code. Thus, we were unable to verify or revise the modeling process used to generate these pooled AUC estimates. According to the IPDmada documentation, the implemented method is based on Zhou *et al.* (13), although this cannot be confirmed directly from the available interface, and we have some doubts about this since the software produces both random- and fixed-effects results. We recognize this as a limitation of the analysis. This is also a common issue in many meta-analyses that depend on user-friendly yet non-transparent software. Nonetheless, it should also be reiterated that our primary analysis was derived from results at the individual patient level, derived from original datasets as continuous variables, which allowed us to calculate the AUC directly as opposed to a summary ROC curve that is typical of standard meta-analyses. Thus, Figs 2 and 4 should be thought of as secondary, exploratory results. Finally, we should mention that while conceptually inconsistent with the reconstructed ROC analysis, these two approaches yielded almost the same results for our data.

Regarding the ‘meta-analysis tables’, the confusion understandably arises from the titles of Tables 2 and 3. The term ‘meta-analysis’ may be generic and not correctly interpreted, as these tables do not present results from a meta-analysis. The tables were intended to present reconstructed diagnostic 2 × 2 tables based on post hoc thresholds derived from the IPD, not pooled estimates from hierarchical models. In this view, the tables represent the summary of reconstructed diagnostic tables (*post hoc* thresholds from IPD) for the calcium stimulation test in females and males.

It is indeed correct that four of five studies were performed in Italy, which may indicate a concentration of research expertise and data in the field of medullary throid cancer (MTC) and calcitonin testing in that context. Despite this, we fully concur that this geographic concentration raises reasonable concerns about overfitting to the local clinical context and local clinical practice, including institutional protocols, assay calibration procedures, and patient management strategies. This local clinical context could limit generalizability to elsewhere; however, it also invoked some consistency in data collection and healthcare protocols, and this limited variability may have improved the internal validity of our pooled analysis. Regardless, we fully agree that future endeavors should aim to include cohorts that are more geographically diverse and improve external validity.

We appreciate the opportunity to respond to the comments of a methodological nature; however, we think it is necessary to answer such methodological critiques within the appropriate clinical context. Medullary thyroid cancer and calcitonin stimulation testing involve unique biological and practical considerations that may not be fully addressed by generalized statistical arguments. We respectfully suggest that applying methodological standards without adequate clinical contextualization may limit their relevance to real-world diagnostic practice.

## Declaration of interest

The authors declare that there is no conflict of interest that could be perceived as prejudicing the impartiality of the work reported.

## Funding

This work did not receive any specific grant from any funding agency in the public, commercial, or not-for-profit sector.
